# Using Theater Testing to Inform Community‐Engaged Cultural Adaptation in Couple and Family Therapy Research

**DOI:** 10.1111/jmft.70116

**Published:** 2026-02-03

**Authors:** Caitlin Edwards, Kendal Holtrop, Andrea Wittenborn

**Affiliations:** ^1^ Human Development and Family Studies Michigan State University East Lansing Michigan USA

## Abstract

Researchers seeking to use community‐engaged methods to culturally adapt a couple and family therapy intervention often face a dilemma of how to gather meaningful feedback from a focal community about adapting an intervention that may be unfamiliar to them. The use of theater testing can provide a beneficial means for solving this challenge. Theater testing is an innovative approach allowing members of the focal population to experience elements of the target intervention to elicit informed and specific feedback. This paper provides a detailed description of theater testing, a set of specific steps for conducting theater testing, and a thorough discussion of the decision points and considerations relevant for applying theater testing to inform cultural adaptation research. We also provide a case example describing the use of theater testing to culturally adapt Emotionally Focused Couple Therapy with members of LGBTQIA+ communities.

Cultural adaptation of therapeutic interventions is necessary due to the worldwide mental health treatment gap (Heim and Kohrt 2019) and the improved efficacy of adapted treatments for diverse populations (Hall et al. 2019; Soto et al. 2018). Theater testing provides a potentially useful methodology for community‐engaged research designed to culturally adapt and tailor interventions. The use of theater testing as a cultural adaptation method can be informed by the six‐step framework of the National Cancer Institute (NCI 2004). This paper will provide an overview of cultural adaptation research in couple and family therapy, describe how theater testing can be applied as a cultural adaptation tool, and present a case example drawn from a recent study on the adaptation of Emotionally Focused Couple Therapy (EFCT) for lesbian, gay, bisexual, transgender, queer/questioning, intersex, and asexual plus (LGBTQIA+) relationships (Edwards et al. 2025b) to highlight salient points for researchers interested in using theater testing.

## 1 Literature Review

### 1.1 Cultural Adaptation of Evidence‐Based Treatments (EBTs)

Cultural adaptation is the systematic alteration of an EBT to account for clients' cultural values and beliefs, enhance relevance, and improve outcomes (Bernal et al. 2009; Lee et al. 2008; Wiltsey Stirman et al. 2019). EBTs contribute to the prevention and treatment of relational and mental health conditions; however, studies often recruit White, heterosexual, cisgender, and middle/higher income samples and then generalize findings to clients with marginalized identities (Spengler et al. 2020). This lack of inclusivity in research maintains mental health disparities, despite the goal of EBTs to reduce treatment inequity and reports that individual therapists routinely, culturally adapt interventions (e.g., Allan et al. 2022; Pepping et al. 2017).

Cultural adaptation broadly encompasses identity (e.g., race, ethnicity, immigration status, ability status, sexual and gender identities, and religious and spiritual orientations) and context (e.g., historical experience, socioeconomic status, geographical setting, and family structure; Holtrop et al. 2025). Several methodological approaches have been used to guide cultural adaptation research. Specifically, methods used to gather consensus and center first‐person experiences to inform cultural adaptation have included Delphi studies (e.g., Lu et al. 2020), focus groups (e.g., J. Parra‐Cardona et al. 2009; Watson‐Singleton et al. 2019), individual interviews (e.g., Ramaiya et al. 2017), and mixed methods studies (e.g., Holtrop et al. 2018; Kohrt et al. 2022).

The primary goal of designing, disseminating, and implementing EBTs is to provide optimal and effective therapeutic treatment (Portney 2020). Intervention researchers often use models to elucidate the process of optimizing an EBT; for example, the Obesity‐Related Behavioral Intervention Trials (ORBIT) model for behavioral treatment development (Powell et al. 2021) describes iterative purpose‐guided phases that direct the collection of evidence needed to support both efficacy and effectiveness of interventions. In the ORBIT model, intervention adaptation often occurs after a nonadapted intervention has been found to be effective, and the intervention is subsequently refined for a new population (Powell et al. 2021).

Intervention adaptation is an inherent part of treatment design (Powell et al. 2021), as interventions may need to be modified to ensure effectiveness in different contexts or with different populations (Movsisyan et al. 2019). Indeed, previous systematic reviews and meta‐analyses indicate that culturally adapted EBTs are more effective than nonadapted EBTs (Hall et al. 2019; Soto et al. 2018).

There are several important decision points when conducting cultural adaptation research, including determining whether cultural adaptation is warranted and which EBT should be adapted (Holtrop et al. 2025). It is also essential to choose a cultural adaptation framework. There are many examples of cultural adaptation frameworks. For example, in the Cultural Sensitivity Framework, Resnicow et al. (1999) describe *surface structure* (e.g., matching intervention materials and methods to observable characteristics of the population) and *deep structure* adaptations (e.g., modification of process elements of an intervention to match the client's worldview). It is also crucial to document all adaptations. One useful tool for documenting the reasons, process, and outcomes of adaptations is the Framework for Reporting Adaptations and Modifications Expanded (Wiltsey Stirman et al. 2019). Finally, pilot and feasibility studies should then be conducted to ensure intervention acceptability, feasibility, proof of concept, efficacy, and effectiveness (Holtrop et al. 2025).

Another key design consideration when pursuing cultural adaptation is the extent to which members of the focal population will be included in this work. Adaptation models vary regarding the need to explicitly include community members (e.g., see Planned Adaptation; Lee et al. 2008 vs. Assessment, Decision, Adaptation, Production, Topical Experts‐Integration, Training, and Testing [ADAPT‐ITT]; Wingood and DiClemente 2008). Yet, many cultural adaptation scholars advocate that members of the focal community should be included throughout the cultural adaptation process to honor their autonomy, account for resilience, and center the multifaceted contexts of the population versus running the risk of focusing on deficits and perpetuating further subjugation (Domenech‐Rodríguez and Bernal 2012; Domenech‐Rodríguez and Wieling 2005). Community‐engaged and participatory approaches are, therefore, widely considered central to the cultural adaptation process (e.g., Holtrop et al. 2025; Substance Abuse and Mental Health Services Administration 2022). One useful approach for strengthening cultural adaptation efforts using community‐engaged research principles is the incorporation of theater testing.

### 1.2 Theater Testing

#### 1.2.1 Background and Overview

Theater testing has roots in advertising research in the 1940s and 1950s, when it was used to assess the persuasiveness of radio commercials (Batra et al. 1996). Theater testing later became a common method for evaluating television advertisements and public service announcements, often as a pretesting technique to aid in formative evaluation efforts (Bartholomew et al. 2006; NCI 2004). It has since been used across fields, such as public health (e.g., Biello et al. 2021), family therapy (e.g., Holtrop et al. 2018), and behavioral health (e.g., Burlew et al. 2023).

Although specific methodological details vary (e.g., see Batra et al. 1996), the traditional format involves inviting members of the target audience to view a draft version of a media product, such as a television commercial (NCI 2004). However, there is typically an element of deception or “camouflage” involved because participants are told they will provide feedback on a television show or other media element, but not the true target of the test, to simulate a real‐world viewing context (Atkin and Freimuth 2013, p. 66; see also NCI 2004). Participants are then exposed to the test commercial in a systematic manner during the decoy television program. Data are collected from participants to measure recall, brand preference, or other variables of interest (Atkin and Freimuth 2013; Batra et al. 1996; NCI 2004).

Theater testing has been described as a form of usability testing (Salazar et al. 2020), an approach meant to evaluate whether a focal product can be used easily and effectively by a target group (Dumas and Redish 1999). A defining characteristic of usability testing is the centering of user experiences by involving them throughout the development and evaluation process and letting user needs guide design decisions (Dumas and Redish 1999). In these ways, theater testing is well‐suited as a method for guiding community‐engaged cultural adaptation efforts.

#### 1.2.2 Theater Testing in Intervention Research

Theater testing has been applied to intervention research in seminal work by Wingood and DiClemente (2008), who developed a systematic framework for adapting evidence‐based interventions for new populations, referred to as the ADAPT‐ITT model. This model was originally developed to guide the adaptation of human immunodeficiency virus (HIV) interventions (Wingood and DiClemente 2008), yet it is broadly applicable to intervention research across a variety of fields and adaptation targets (e.g., Holtrop et al. 2018). ADAPT‐ITT sets forth a series of eight phases to guide the adaptation research process: (1) assessment, (2) decision, (3) adaptation, (4) production, (5) topical experts, (6) integration, (7) training, and (8) testing.

Theater testing is an integral part of the ADAPT‐ITT model that takes place during Phase 3 (Wingood and DiClemente 2008). To inform the adaptation efforts, members from the new focal population are recruited for a testing group, during which they participate in key segments of the original intervention. In this way, theater testing is an active pretesting approach that simulates what participants would experience during the intervention. Fostering an in vivo experience of the focal intervention strengthens the adaptation process and constitutes a salient strength of this method (Wingood and DiClemente 2008). Agency staff and other stakeholders may also be invited to attend and observe the theater test. After each intervention segment is delivered, attendees respond to brief surveys designed to elicit feedback on the appropriateness of the material, critiques of the original intervention elements, and suggestions for intervention additions relevant to the new focal population. Facilitators then use the survey responses to lead a group discussion about the intervention elements, first with participants from the focal population and subsequently by inviting input from the agency staff and stakeholders. Data are analyzed to identify common themes regarding how the original intervention should be adapted to better meet the needs of the new focal population (Wingood and DiClemente 2008). It is important to note that theater testing can result in both surface structure and deep structure adaptations (see Resnicow et al. 1999).

#### 1.2.3 Applications of Theater Testing

Theater testing has been used across a number of studies seeking to develop or adapt various interventions. A sizeable body of this work has focused on HIV research (e.g., Burlew et al. 2023; Lopez et al. 2025), likely as a result of Wingood and DiClemente's (2008) influence on this field. Important advancements have also been made in extending theater testing to behavioral and mental health interventions, in large part due to recent applications of the ADAPT‐ITT model in these areas. For example, theater testing was used to strengthen participatory research efforts to support psychological health and well‐being among Native American Head Start teachers in the United States (Wilson et al. 2023) and to leverage a peer‐delivery model to enhance mental health services for youth in Sierra Leone (Freeman et al. 2024). Notable applications of theater testing have been carried out in couple and family intervention research as well. The most salient body of this work has focused on the adaptation of parenting interventions for different populations, such as parents aging out of the child welfare system (Holtrop et al. 2018), Hmong Americans (Zhou et al. 2018), and caregivers of LGBTQ+ foster teens (Salazar et al. 2020). Additionally, Edwards et al. (2025a) used theater testing to adapt an evidence‐based couple intervention for LGBTQIA+ relationships. Despite such efforts, the application of theater testing remains underutilized in couple and family intervention research.

## 2 Application of Theater Testing to Cultural Adaptation Research

Cultural adaptation frameworks are important for guiding overall study design, but methodologies for carrying out specific stages of cultural adaptation research are less well‐developed. Theater testing is a promising method to use during the cultural adaptation process, particularly for scholars committed to using a participatory research approach. In this section, we will explain how theater testing can be applied to cultural adaptation research using the six theater testing steps described by the NCI (2004): (1) plan the theater test, (2) develop the questionnaire, (3) recruit respondents, (4) prepare for the theater test, (5) conduct the theater test, and (6) analyze the theater test. To more fully illustrate this process, we will also present a case study, based on work by Edwards et al. (2025a), depicting the application of theater testing to adapt a couple therapy intervention for LGBTQIA+ relationships.

### 2.1 Plan the Theater Test

#### 2.1.1 Theater Testing Guidelines

When planning the theater test, researchers must determine the purpose of the study, their timeline and budget, who to recruit, and the pretest location (NCI 2004). The planning stage is important for ensuring a theater test will meet the study objectives and be conducted feasibly, given the available resources. A central benefit of theater testing is that it allows potential users to gain exposure and provide responses to focal material (Batra et al. 1996). In this way, theater testing is a critical tool for determining if intervention components are engaging, understandable, and culturally relevant to potential users (Bartholomew et al. 2006).

#### 2.1.2 Theater Testing in Cultural Adaptation Research

Theater testing applied to cultural adaptation research involves understanding how an intervention should be tailored to meet the needs of the target population *and* how the target population's lived experience can inform this tailoring (Wingood and DiClemente 2008). Therefore, identifying the focal population and selecting the target intervention are important components of the planning stage.

Cultural adaptations are indicated when marginalized populations are experiencing health disparities, engagement constraints in accessing existing treatments, and/or culturally specific risk factors (Holtrop et al. 2025). The presence of such factors may help identify a focal population for cultural adaptation efforts, but only if representatives of this population are included in the decision‐making process and affirm this decision (Holtrop et al. 2025). It is also beneficial for members of the cultural adaptation research team to have a connection with the chosen population (Gonzalez et al. 2021), as a shared connection can foster trust (Andoh‐Arthur 2019). Knowledge of the chosen intervention can assist with cultural adaptation, as the researcher is better prepared to evaluate both surface structure (e.g., materials provided to the focal population) and deep structure (e.g., integration of cultural factors) adaptations (Resnicow et al. 1999).

In line with best practices, the target intervention should have a body of research evidence establishing its effectiveness (Domenech‐Rodríguez and Bernal 2012; Holtrop et al. 2025). Selection of the target intervention from among the available EBTs should be based on the relevance of the intervention for the focal population and their existing mental health needs (Barrera et al. 2013). Goodness of fit is a key aspect of this decision, as cultural relevance, acceptability, capacity, and feasibility are all critical considerations (Domenech‐Rodríguez and Bernal 2012; Holtrop et al. 2025; Wingood and DiClemente 2008).

Additional planning considerations can then build from these core determinations. Researchers should account for participant access to transportation, travel time and costs, as well as the ability to use technology as relevant. Theater testing traditionally occurs in person (NCI 2004; Wingood and DiClemente 2008), so researchers should account for participant access to transportation, travel time, and costs involved in attending. Researchers must identify a location with enough space to accommodate the theater test. At the same time, researchers must be sensitive to safety considerations and understand potential locations where participants feel comfortable (e.g., see Baumann et al. 2011). For theater testing occurring online, it is important to pilot test the data collection platforms and other technology to ensure ease of use for the focal population as well as establish the general functionality of the procedures. Researchers should plan to adequately compensate participants and ensure sufficient time for data collection.

#### 2.1.3 Case Example

The study described in this case example aimed to provide first‐person recommendations of how EFCT (Johnson and Greenberg 1985; Johnson 2019) could be adapted to account for the unique lived experiences of LGBTQIA+ identifying individuals in romantic relationships. EFCT is an empirically supported treatment for relationship distress that has demonstrated significant positive outcomes (Doss et al. 2022; Spengler et al. 2024), including relationship distress recovery (Johnson et al. 1999). There is evidence that EFCT therapists routinely work with LGBTQIA+ relationships (Allan et al. 2022; Edwards et al. 2025a), and conceptual articles (Allan and Johnson 2017; Hardtke et al. 2010) describe the use of EFCT with gay and lesbian relationships. However, only limited research to date has documented the use of EFCT with LGBTQIA+ relationships (Edwards et al. 2025b). As LGBTQIA+ identifying individuals in romantic relationships have less access to EBTs compared with their heterosexual counterparts (Alencar Albuquerque et al. 2016), it is essential that EBTs are adapted to account for these clients' unique lived experiences.

EFCT was chosen for adaptation for two reasons. First, EFCT is one of the most heavily researched and effective models of couple therapy (Doss et al. 2022). Despite this, EFCT research to date has historically privileged White, heterosexual, and cisgender identities and relationships (Spengler et al. 2024). Although contemporary scholarship has begun to remedy this disparity (see Allan et al. 2022; Guillory 2021; Hattori 2014; Nightingale et al. 2019; Tseng et al. 2025), the limited inclusion of diverse study samples in EFCT research, such as LGBTQIA+ identifying individuals, represents a significant limitation if this EBT is going to be generalizable across populations. Indeed, to date, no empirical process (e.g., ADAPT‐ITT) for adapting EFCT to account for client identity and/or cultural context has occurred. Second, attachment strategies and behaviors are impacted by sociocultural context (Dunbar et al. 2022). For example, Dunbar et al. (2022) found that Black mothers who prepare their children to experience bias with a combination of high emotional support and moderate suppression of their distress response experience secure attachment with their children, unlike what has been shown in White families.

In attending to additional planning considerations, we elected to conduct the theater testing online for several reasons. First, we planned to recruit globally, as EFT centers and communities exist in 39 countries. Second, the training videos we showed were easily accessed online. Third, online meetings were more accessible for participants.

### 2.2 Develop the Questionnaire and/or Interview Questions

#### 2.2.1 Theater Testing Guidelines

In the early stages of designing a pretest, it is also important to develop the questionnaire or interview questions that will be administered (NCI 2004). To do this, researchers must be clear about their operational objective and the specific variables they wish to assess; successful theater testing depends on the relevant variables being identified and measured well (Batra et al. 1996). In traditional theater testing, questions are asked about the program viewed to elicit participant reactions (e.g., things they liked and did not like about the program), to assess whether participants can identify and recall the main ideas presented in the focal material, and to identify what participants thought and felt while viewing the focal material; questions specific to the intended audience may also be included (NCI 2004).

#### 2.2.2 Theater Testing in Cultural Adaptation Research

Cultural adaptation studies are often concerned with the acceptability and appropriateness of the intervention (e.g., Zubieta et al. 2020), and theater testing is particularly well‐suited for assessing these constructs. For example, when developing study questions, participants could be asked what they liked (and did not like) about the focal intervention, what ideas or strategies they found most relevant (and least relevant), and whether they see themselves using any of the tools described in the intervention. Questions can also be tailored to certain elements of the focal material and characteristics of the intended audience (NCI 2004). This enables cultural adaptation researchers to get feedback on specific components of the focal intervention (e.g., online delivery, experiential activities, interactions with group members, and receiving a homework assignment), as long as components are administered as part of the theater test, and to expressly inquire about participants' experiences based on their membership in the focal population.

This decision‐making process will involve making determinations regarding whether quantitative data, qualitative data, or some type of multimethod or mixed method strategy will best achieve the study's aims. Traditional theater testing norms involve the use of multiple methods, such as quantitative surveys and focus groups (NCI 2004). The theater testing procedure described by Wingood and DiClemente (2008) involves administering a survey to participants with both closed‐ended and open‐ended questions and then using the survey responses to guide a subsequent group discussion about the focal intervention. Moreover, prior cultural adaptation studies using theater testing have used diverse approaches (e.g., Holtrop et al. 2018; Lopez et al. 2025; Singer et al. 2024); therefore, there is ample opportunity to use various approaches to data collection when conducting theater testing.

While developing the questionnaire, some additional considerations also apply. First, it is critical for researchers to consider participants' potential levels of comfort/discomfort in discussing certain information, particularly among participants holding marginalized identities. Second, participants may be keen to provide feedback and likely to venture into areas that are outside of the scope of the study. To prevent such drift, questions should be specific. Instead of asking, for example, if participants found the intervention to be helpful, questions should focus specifically on the relevant elements of the intervention (e.g., content, video examples, teaching style, and group discussion). If a semistructured interview is being used for data collection, these specific questions could be included in the interview guide as follow‐up/probing questions to help redirect the interview as needed (see Edwards et al. 2025a). It is also highly important to pilot test the questionnaire prior to starting data collection so that questions prone to irrelevant responses can be improved (NCI 2004). Third, participants may benefit from guidance about the specific aspect(s) of their multifaceted identity that represent the focus of the cultural adaptation efforts. For instance, a theater test to adapt a parenting intervention for Latinx caregivers would want to focus data collection efforts on the experiences of participants most related to their membership in the focal group, Latinx caregivers, and less on other identities. Providing cues to prompt responses from this focal perspective can be a useful strategy for obtaining the most relevant data (e.g., “What parenting strategies presented were, or were not, relevant to your needs as a Latinx caregiver?”). This is another area in which pilot testing is highly important.

#### 2.2.3 Case Example

Prior to developing the questionnaire, we met to discuss and define the operational objectives of the study. Broadly, our goal was to increase the cultural relevance of EFCT for LGBTQIA+ relationships. Specifically, we wanted to understand how EFCT therapists could increasingly integrate the unique lived experiences of LGBTQIA+ people into EFCT interventions. For example, we aimed to understand how participants could benefit from integrating experiences of minority stress (e.g., Frost and Meyer 2023) into the negative interaction cycle (i.e., harmful interaction patterns which reinforce negative attributions and relational disconnection in relationships; Johnson 2019).

We collected qualitative data through focus groups. Focus groups have a history of being used in cultural adaptation research (e.g., Scott and Rhoades 2014) and activist contexts (Kozol 1985; Madriz 2003) and provide several methodological strengths, such as providing a balance between breadth and depth of information and allows for a deeper understanding of co‐constructed meanings (Krueger and Casey 2015). Specifically, we asked participants to view segments of an EFCT training video depicting work with the same sex couples and then provide their feedback using a structured interview.

To develop the interview protocol, we followed the focus group questionnaire development model outlined by Krueger and Casey (2015). We began by identifying the key questions that should be asked after viewing each video segment. For example, one key question focused on how to integrate discussion of common gay male experiences into EFCT's focus on identifying challenges to connection when tracking the negative interaction cycle. Specifically, we asked: “Sometimes in therapy, we talk about challenges to connection, for example, in this clip you see Tim talk about how his experience of being a gay man and how this leads to the need to be fiercely independent. How did the therapist talk about the challenges of connection as related to Tim and Andrew's identities as gay men? What more could be done?” We then developed the general, opening questions, which focused on priming participants to think about their experiences of EFCT from the lens of their identities as members of LGBTQIA+ communities. For example, the first opening question was: “How would you describe your experience of EFCT from the lens of your LGBTQIA+ identity.” These questions were designed to both evoke the salient aspect of identity we desired participants to focus on and to point to a specific aspect of EFCT we identified as modifiable.

All questions were developed with experienced EFCT researchers and clinicians who identified as members of LGBTQIA+ communities to ensure that questions captured specific elements of EFCT and were culturally relevant. Questions were iteratively revised over time to ensure that they were both comfortable and appropriate for participants. After initial question development, we then pilot tested the interview protocol with LGBTQIA+ identifying scholars.

The first author also met with each participant individually prior to the focus group to discuss the potential lack of comfort participants could experience due to historical experiences of discrimination in the context of research and the sensitive nature of data collection. They thoroughly discussed confidentiality and privacy with each participant. They also encouraged participants to email them about any thoughts the participants did not have sufficient time to share during the theater test and/or any thoughts they may have been too uncomfortable to share in the group setting.

### 2.3 Recruit Respondents

#### 2.3.1 Theater Testing Guidelines

Another important step in theater testing is recruitment (NCI 2004). The advertising literature describes the importance of ensuring that the target market is clearly defined so that respondents who represent that population can be recruited for the theater test (Batra et al. 1996). That is, to ensure a theater test provides a valid indicator of usability, participants who represent real potential users need to be included (Dumas and Redish 1999). The original theater testing procedures, which relied on broad exposure and questionnaire data, often used large sample sizes in the hundreds (Batra et al. 1996), though more recent guidelines suggest fewer participants are sufficient (e.g., *n* = 50; NCI 2004). When considering issues of recruitment, researchers should also determine who will support recruitment efforts (e.g., recruitment agency or partnership with community organization) and how participants—and possibly recruitment partners as well—will be incentivized for their efforts (NCI 2004).

#### 2.3.2 Theater Testing in Cultural Adaptation Research

Recruitment efforts in a cultural adaptation study must also begin with clearly delineating the focal population (Heim et al. 2021). Researchers must then determine from whom they will collect data to inform their adaptation efforts. While cultural adaptations can be guided by a variety of sources, such as cultural informants (e.g., Abi Ramia et al. 2018), topical experts (e.g., Edwards et al. 2025b), scholarly experts, and extant literature (e.g., Pentel et al. 2020), theater testing is specifically meant to collect data from members of the intended audience (Batra et al. 1996; Dumas and Redish 1999; Wingood and DiClemente 2008). This feature is an important strength of using a theater testing approach for cultural adaptation. Without a pretesting process in place, scholars have cautioned that, “people who have been developing complex products but have never systematically watched users set up, learn, or use those products find watching a usability test fascinating, shocking, sometimes humiliating and painful, but invariably eye‐opening” (Dumas and Redish 1999, p. 89). Therefore, intended users (e.g., potential participants, service providers, or agency personnel) should be participants in the theater test. Prior to recruiting, the characteristics of the intended participants should be operationalized into inclusion and exclusion criteria.

Culturally relevant and responsive community‐based engagement, initiated early in the research process, is critical for supporting recruitment and retention of individuals from marginalized communities (Cunningham‐Erves et al. 2023). Such collaboration increases researchers' understanding of community priorities, ensures cultural relevance, and creates trust (London et al. 2022). Many focal populations in cultural adaptation research hold marginalized identities or statuses that may put them at risk. For instance, Baumann et al. (2011) described how Latinx caregivers in their cultural adaptation project were negatively impacted by anti‐immigration sentiment and ongoing raids by U.S. Immigration and Customs Enforcement. While marginalized populations have sometimes been labeled *hard to reach*, scholars argue these populations are indeed reachable if conventional research methods are adjusted to respond to the needs and contextual realities of these groups (e.g., Aliyas et al. 2023). Culturally appropriate recruitment strategies, such as those suggested in the cultural adaptation research literature, should therefore be applied in theater testing. Examples include using word‐of‐mouth referrals from prior participants (J. R. Parra‐Cardona et al. 2014), in‐person discussions with healthcare providers and community gatekeepers (Gonzalez et al. 2021), or culturally tailored social media marketing (Simenec et al. 2023).

Furthermore, in contrast to the large sample sizes often targeted in advertising‐based theater testing (see Batra et al. 1996), applications to intervention research typically focus on smaller samples. Wingood and DiClemente (2008) specify a sample of *n* = 15. Other cultural adaptation studies using theater testing have likewise recruited smaller samples ranging from 8 (Breslow et al. 2024) to 112 (MacEntee et al. 2022). These smaller samples are not intended to be broad and representative; instead, smaller, targeted samples in theater testing research are meant to purposefully include individuals holding the identities of the focal population and garner an in‐depth understanding of their lived experiences.

Ethical research practices also stipulate equitable compensation for participants (Różyńska 2022), as fair compensation enhances study participation. Inadequate compensation can exploit economically disadvantaged individuals, create barriers to participation, and potentially undermine the diversity and inclusivity of research findings. Given the historical injustice experienced by marginalized communities, it is especially important to provide adequate payment for participation (McCracken 2020). Moreover, fair payment acknowledges participants' time and effort, fosters trust, and encourages informed, voluntary participation (Różyńska 2022). Ethical guidelines emphasize that compensation should be reasonable and not coercive, ensuring that participants are not unduly influenced by financial incentives (Różyńska 2022).

#### 2.3.3 Case Example

The LGBTQIA+ acronym denotes a wide range of identities and experiences; this inherently meant the population we selected was both highly variable and clearly defined as sexual and/or gender minorities. To access LGBTQIA+ identifying EFCT clients, we planned to recruit EFCT therapists functioning as community gatekeepers (i.e., mediators who facilitate trustful access to study settings and participants; Andoh‐Arthur 2019). This necessitated several recruitment methods. We advertised the study on the queer EFCT listserv, the International Centre for Excellence in Emotionally Focused Therapy (ICEEFT) listserv, and queer EFCT Facebook groups. We contacted the leaders of EFCT centers and communities and asked them to distribute study information, as well as emailed all therapists from English‐speaking countries listed on the ICEEFT directory. The first author also leveraged their EFCT connections to contact EFCT therapists who work with LGBTQIA+ communities.

Participants were included in the study if they (a) self‐identified as a member of LGBTQIA+ communities, (b) completed at least six EFCT sessions with a partner of more than 1 year, and (c) were at least 18 years old. Participants were excluded if they were experiencing active psychosis or intimate partner violence. Participants who had experienced suicidal ideation with intent within 1 month of the study were also excluded and provided crisis resources. To ensure adequate compensation, we provided each participant with a $100 gift card.

To ensure that participants were able to understand the questions and provide relevant feedback, the first author conducted a pretest with five LGBTQIA+ identifying scholars. These scholars provided feedback on the structure of the questions, the flow of the study, and the aspects of EFCT that were demonstrated.

### 2.4 Prepare for the Theater Test

#### 2.4.1 Theater Testing Guidelines

Prior to conducting the theater test, additional preparations must be made to ensure successful administration. Researchers must make decisions about the material that will be featured in the theater test. For example, will participants be shown a draft of the material at an earlier stage of completion or something more closely resembling the finished product (Batra et al. 1996)? And how much exposure to the focal material is needed to provide meaningful results (Batra et al. 1996)? Researchers must also ensure the featured material is ready for presentation. This includes ensuring audiovisual materials are prepared, all equipment is functioning properly, and participants are able to see and hear the content (NCI 2004). Researchers should also ensure that all theater testing personnel understand their roles and have made arrangements to attend. To aid in preparing for the pretest, holding a rehearsal session prior to the first theater test to try out the procedures and troubleshoot any identified problems is recommended (NCI 2004).

#### 2.4.2 Theater Testing in Cultural Adaptation Research

There are several decisions researchers must make about the intervention content they want to demonstrate. First, at which stage of cultural adaptation will the intervention be presented? Wingood and DiClemente (2008) describe using the original intervention in their theater testing procedure, while others (e.g.,MacEntee et al. 2022) have demonstrated adapted versions. Depending on the stage of the research and the existing cultural adaptations of the chosen intervention, each method answers different research questions. Researchers looking to understand what and how to adapt an intervention for a specific cultural group could show the original intervention and then adapt it based on participant feedback. For example, Biello et al. (2021) asked men who have sex with men (MSM) for feedback on the MyChoices app prototype prior to its adaptation. On the other hand, researchers wanting to gauge the preliminary acceptability of an adapted intervention may choose to demonstrate previously tailored content. For instance, MacEntee et al. (2022) used key informant interviews and focus groups to assess stakeholder perspectives on street‐connected youth's access to services and how current interventions could be adapted to increase accessibility.

Researchers also need to decide which aspects of the intervention will be shown during the theater test (NCI 2004). It is important for researchers to understand which aspects of the model may be important to the focal group. There is also a need to demonstrate the aspects of the intervention that are modifiable, while maintaining the core components of the model, in line with best practices in cultural adaptation research (Bernal et al. 2009; Holtrop et al. 2025; Resnicow 2000). Wingood and DiClemente (2008) specify demonstrating the core components of an intervention so that participants have a strong understanding of the purpose and process of the intervention. As part of these decisions, researchers must also consider whether all selected intervention content should be presented at one time or if the material should be presented in segments, with data collection taking place at multiple points during the theater test.

Another consideration is how researchers will provide the demonstration. Original theater testing research focuses on watching audiovisual media (NCI 2004), while Wingood and DiClemente (2008) discuss the benefits of experiential delivery of an intervention. Overall, aside from a live, in vivo demonstration, there are many creative options for exhibiting an intervention. For example, MacEntee et al. (2022) elected to develop films for their theater test to demonstrate the role of peer navigators in their adapted intervention. Kazemi et al. (2018) conducted a live demonstration of an app designed to decrease a user's hazardous drinking, while the MSM in the study by Biello et al. (2021) downloaded an HIV prevention app and provided feedback in real time during use.

#### 2.4.3 Case Example

As part of our preparation for the pretest, we decided to use video segments from the EFCT training DVD, *The EFT Path to Secure Connection: Working Successfully with Same‐Sex Couples* (Reel Concepts for Susan Johnson Inc. 2011), to depict the intervention. At the time of this study, the adaptation of EFCT for LGBTQIA+ individuals and relationships was in the early stages. Therefore, we elected to demonstrate an original, manualized implementation of EFCT (i.e., an available version as relevant as possible to our focal population) rather than piloting a nonmanualized adaptation of EFCT for the focal population. This was justified given the stage of the research and our research questions.

Our preparation activities also included selecting two segments of the EFCT intervention to demonstrate during the theater test. To inform this decision, in line with established guidelines in cultural adaptation research (Bernal et al. 2009; Holtrop et al. 2025), we identified the core processes of EFCT that should remain unaltered (e.g., tracking the negative cycle, the EFT Tango; Johnson 2019) as well as elements that could be adapted to account for the unique lived experiences of members of LGBTQIA+ communities. Identifying which intervention segments to demonstrate was a difficult process because EFCT work is dynamic, nonlinear, and evolves over time; therefore, it is not easily encapsulated in a brief intervention segment. In consultation with EFCT therapists and researchers, we elected to demonstrate 20 min from early in treatment (i.e., stage one) and 20 min later in treatment (i.e., stage two) with the hope that participants would not lose interest while simultaneously viewing enough of the session to make informed comments. We chose each segment because it demonstrated fidelity to the EFCT model and highlighted unique aspects of the gay male community (e.g., the struggle to emotionally connect because of the need to be fiercely independent) and the lesbian community (e.g., lesbian bed death). Furthermore, each segment demonstrated a key step in each stage of EFCT.

We decided to conduct the theater test using videos rather than live demonstrations for several reasons. First, the use of video is in line with early theater testing research (NCI 2004). Second, the use of existing videos ensured that the intervention would be demonstrated with fidelity. Third, the use of videos was more feasible and resource‐efficient than scheduling live demonstrations, especially since we administered multiple theater testing sessions.

To prepare for the theater test, the first author pilot tested all audiovisual materials. We subsequently altered how the study was introduced (i.e., we indicated that the training tapes were made in the mid‐2000s and would likely not represent a “modern” queer experience) and added a 5‐min break in between the discussion of the stage one tape and the showing of the stage two tape. Even with these efforts, we did experience technological challenges administering the video that we had to subsequently resolve, further underscoring the importance of preparing for the theater test using conditions that reflect the true testing environment as closely as possible.

### 2.5 Conduct the Theater Test

#### 2.5.1 Theater Testing Guidelines

To conduct a successful theater testing session, researchers should foster an experience that combines showing warmth and courtesy to participants by carrying out an organized and structured protocol. Guidelines (NCI 2004) suggest helping participants to get settled, starting on time, and beginning by expressing gratitude to participants for attending, introducing the researcher(s), and setting a positive expectation for the theater test. Participant reactivity is a concern during theater testing, such that individuals may act or respond to questions differently in the testing environment than they would under normal conditions (Batra et al. 1996). Although deception has been suggested as a means for averting reactivity, this has ethical implications (Batra et al. 1996). Emphasizing to participants that you are interested in their perceptions and that there are no right or wrong answers can be useful in this regard (NCI 2004); unobtrusive observational data collection (with participant consent) may also be effective (Batra et al. 1996). Overall, all reasonable efforts that help participants feel comfortable and at ease during the session are important while conducting the theater test.

Next, the researchers will distribute the data collection instruments and provide any relevant instructions up front (e.g., discourage talking during the demonstration; NCI 2004). Participants should then be exposed to the test material. Researchers must ensure the material is delivered as intended and be prepared to troubleshoot any issues that arise. They should also remain attentive to making sure participants remain engaged and follow the instructions provided (NCI 2004). During the demonstration, participants should interact with the material in ways that are as real as possible (Dumas and Redish 1999). After the test material has been presented, researchers must ensure all data have been received (e.g., questionnaires collected, online surveys submitted; NCI 2004). At the conclusion of the theater testing session, it is important to thank participants for their time and effort and provide any prearranged compensation. Theater testing guidelines recommend the session last no longer than 1 h and 15 min (NCI 2004).

#### 2.5.2 Theater Testing in Cultural Adaptation Research

Wingood and DiClemente (2008) recommend inviting both the focal group as well as key stakeholders to participate in the theater test. For example, MacEntee et al. (2022) included healthcare providers, community service providers, and the focal youth in a series of theater tests. Additionally, theater testing guidelines (Wingood and DiClemente 2008) suggest having participants complete brief surveys containing both closed‐ and open‐ended questions to elicit reactions regarding the suitability of the intervention for the focal group. Ideally, the questions would prompt participants to appraise the material, content, and delivery of the intervention as well as identify other materials and/or activities that would increase cultural relevance and efficacy. The survey responses then prompt group discussion between key stakeholders and the focal population, focused on developing ideas for intervention adaptation (Wingood and DiClemente 2008).

Researchers must decide if it is more effective to collect all data at the end of the theater test or at multiple points between segments of the intervention. Thus, researchers should consider the complexity of the chosen intervention, the capacity of participants, and the aims of the research. Other considerations include how to collect high‐quality and relevant data that address the research question rather than other elements of the intervention (e.g., therapist factors), how to set up the data collection process to be successful, and how to redirect and focus participants. Therefore, interview protocols and surveys should be carefully developed and pilot tested prior to implementation, and researchers should be well trained in data collection processes.

Researcher observations are also a potentially important data source when conducting theater testing (Dumas and Redish 1999). Therefore, researchers should both observe and record participant verbal and nonverbal behavior. These observations have the potential to inform the delivery of the chosen intervention, as cultural norms may influence who provides the intervention as well as the process of delivery (Soto et al. 2018). For example, Biello et al. (2021) observed participants interacting with the MyChoices app, which influenced modifications to the app in the hopes of increasing PrEP usage for MSM.

#### 2.5.3 Case Example

We conducted eight theater testing groups in total, each lasting between 105 and 142 min (*M* = 122). Since research on the adaptation of EFCT for LGBTQIA+ relationships using topical experts had already occurred (Edwards et al. 2025a), the theater testing groups solely included individuals from the focal population. As soon as everyone joined the virtual meeting, we introduced the study and asked participants to introduce themselves. We then described the purpose and nature of the study and reiterated confidentiality. Next, we outlined focus group and theater testing procedures as well as introduced group norms (e.g., taking turns when speaking) to guide the conversation. We also discussed strategies for managing feelings of distress, including taking a break, turning the camera off, and/or debriefing with the researcher after the focus group.

After the introduction to the focus group, we followed the semistructured interview protocol and asked participants about their general experience of EFCT, if they had discussed experiences of minority stress with their relationship therapist, and how their therapist created safety in therapy in reference to their identity as sexual and/or gender minorities. Examples of probing questions included: “How did you find your EFCT therapist?” and “How have experiences of discrimination impacted your romantic relationships?” As part of the interview protocol, participants were prompted to think specifically about their LGBTQIA+ identities in relation to these research questions.

Next, we showed the two selected segments of the EFCT intervention video. The first segment involved tracking the negative interaction cycle in stage one with a gay male couple. We then asked the following questions: “We know that knowledge of identity increases safety in therapy. How do you experience this therapist creating safety for this couple? What more could she do?” and “sometimes in therapy, we talk about challenges to connection, for example, in this clip you see Tim talk about how his experience of being a gay man and how this leads to this need to be fiercely independent. How did the therapist talk about the challenges of connection as related to Tim and Andrew's identities as gay men? What more could she do?” There was a 5‐min break between this discussion and viewing the stage two segment, which focused on withdrawer re‐engagement with a lesbian couple. After segment two, we asked about general reactions, what participants noticed about the therapist's discussion of lesbian sex, and the following: “Sometimes in therapy, we talk about how fear impacts deeper connection in relationships. For example, in the clip, you saw Janey talk about how her fear blocks her from receiving care from her partner, Kelly. How do you think the therapist navigated talking about fear given that fear is a common lived experience of members of LGBTQIA+ communities?”

When participants deviated from the topic focus, the first author redirected them back to the questions. For example, participants mentioned discussing past traumas in individual but not couple therapy. The first author validated these experiences and refocused participants on their experiences in EFCT. We also focused on observing and recording participant verbal statements and nonverbal behaviors.

### 2.6 Analyze the Theater Test

#### 2.6.1 Theater Testing Guidelines

The theater testing literature suggests data analysis takes place in two steps: tabulate responses and look for patterns (NCI 2004). When analyzing qualitative data, using a specific qualitative analytic framework can aid in the systematic search for patterns or themes. If mixed, multiple, or quantitative methods are used, research questions are often more easily answered using descriptive data rather than inferential statistics (Dumas and Redish 1999; NCI 2004). When analyzing the data, researchers may want to consider (NCI 2004): (a) what was learned, (b) whether the reaction from respondents was favorable, (c) if the intervention material demonstrated fulfilled the desired objectives, (d) the strengths and weaknesses of the demonstrated material, and (e) what results are most salient. Answering these questions allows researchers to understand the experiences of participants and make determinations regarding whether the material is appropriate and effective or needs further revision (NCI 2004).

#### 2.6.2 Theater Testing in Cultural Adaptation Research

Analyzing the data from a theater test to inform cultural adaptations involves a few unique decision points. As theater testing data can be voluminous, a primary task during data analysis is to reduce the amount of data. Therefore, analysis involves returning to the intent of the study and excluding superfluous and irrelevant information. Cultural adaptation researchers must decide which data are relevant to the study objectives and which can be set aside without compromising study validity or rigor. In addition, cultural adaptation researchers must seek to understand the data in relation to issues of intervention fidelity. In general, cultural adaptations of interventions preserve core intervention components (Holtrop et al. 2025; Resnicow et al. 1999; Wiltsey Stirman et al. 2019), unless relevant data strongly suggest otherwise (Wiltsey Stirman et al. 2019). Theater testing participants may provide recommendations that are not consistent with the core components of the model (Wingood and DiClemente 2008) or suggest modifications that would reduce model fidelity (Wiltsey Stirman et al. 2019). Cultural adaptation scholars must carefully consider the weight of the empirical evidence regarding intervention core components and mechanisms of change, alongside participant perceptions, when deciding whether to make fidelity consistent versus fidelity inconsistent changes to the intervention based on theater testing data. It is incumbent upon cultural adaptation researchers to modify the intervention in ways that are consistent with model fidelity or provide justification that changing core components of an intervention would dramatically improve cultural relevance and/or not undermine treatment efficacy (Wiltsey Stirman et al. 2019).

#### 2.6.3 Case Example

As we prepared to analyze our theater testing data, it was difficult to discern what information was meaningful and related to the research question and what information was meaningful but extraneous to the current project. We therefore spent additional time considering what data were relevant to the modification of EFCT and what data were unrelated to the research question. For example, many participants discussed how their own therapist makes adaptations to the model; indeed, one participant discussed how important it was that their therapist discussed how racism impacts their negative interaction cycle. While this information is important, it was outside the scope of the current research project.

Some participants emphasized recommendations that were not in line with the core components of EFCT. For example, two participants strongly advocated for therapists to provide psychoeducation about healthy relationships and behaviors that could be considered “normal” in romantic relationships. However, EFCT is an experiential model, and does not explicitly incorporate psychoeducation (Johnson 2019). As such, adding this to the steps and stages of EFCT would be inconsistent with model fidelity. Other participants suggested that EFCT therapists could specifically ask how the experience of fear related to LGBTQIA+ identity impacted the negative interaction cycle, which aligns with the assessment and evocative questioning process that is core to EFCT; this adaptation would be considered fidelity consistent, as it focuses on integrating external experiences into the negative interaction cycle.

In the end, our theater test data provided several recommendations for tailoring EFCT, including surface structure adaptations meant to better match the intervention with LGBTQIA+ relationship characteristics (e.g., including a session specific to discussing how gender and sexual identity inform relationship dynamics) and deep structure adaptations meant to better align the intervention with the lived experiences of LGBTQIA+ individuals (e.g., discussing how identity‐based experiences of fear impact the negative interaction cycle). The full set of recommended adaptations is presented and discussed in Edwards et al. (2025a).

## 3 Discussion

For couple and family therapy researchers engaged in cultural adaptation research, theater testing offers a useful approach for engaging members of the focal community in the adaptation process. In this paper, we have described the planning, execution, and evaluation of theater testing for cultural adaptation research using the NCI (2004) six‐step framework. When using theater testing, it is essential to start with a planning stage that includes clarifying the purpose of the adaptation, the focal population, the timeline, budget, and theater testing location (step 1). Additionally, researchers should ensure they are best assessing all relevant variables when developing the questionnaires or interview questions (step 2) and give careful consideration to recruiting the focal population (step 3). All procedures and materials should be prepared in a manner that is understandable and accessible for participants (step 4). Ideally, when conducting the theater test, researchers will build rapport with participants and ensure the intervention is demonstrated in a “real world” manner (step 5). Finally, when analyzing the data, it is essential to ensure the analytic methods allow researchers to assess strengths and weaknesses, as well as all relevant information related to intervention adaptation (step 6). It is also important to note that the steps we have described are not necessarily linear, and fluid movement can occur between steps, especially during the early stages. Subsequently, the adapted intervention should be subjected to fidelity testing and further empirical evaluation, such as efficacy and effectiveness trials, in line with the ORBIT model (Powell et al. 2022).

While the case study described provides a strong example of the use of theater testing to inform cultural adaptation efforts, there are modifications that we recommend that align with the NCI (2004) framework. First, we believe the video segments used may have been too lengthy. Each segment was approximately 20 min, which resulted in theater testing averaging 122 min, longer than the 75–90 min recommended in the literature (NCI 2004; Wingood and DiClemente 2008). Second, it may have strengthened data collection to supply participants with multiple ways of providing feedback, such as the use of a questionnaire or a quantitative survey. Finally, although our study took place online, participants reported struggles paying attention and engaging online, indicating that a physical location and in‐person demonstration may have improved data collection.

Theater testing is a critical method of conducting community‐engaged research. Broadly, the goals of community‐engaged research are to center and elevate underrepresented communities as well as develop and disseminate culturally appropriate interventions that engender policy change and aid in the translation of research findings to community‐based implementation (Oetzel et al. 2018). Community‐engaged research is inherently collaborative, in which trust, partnership, and resources are shared to enhance community‐based outcomes (Oetzel et al. 2018). Theater testing specifically provides a relevant approach for using community‐ and focal population‐generated data to guide cultural adaptation and help ensure the cultural relevance of an intervention.

We have demonstrated that theater testing has broad application in cultural adaptation research. This flexible application also speaks to the types of studies that can utilize theater testing, ranging from dissertation research (e.g., Edwards et al. 2025a) to nationally funded studies (e.g., MacEntee et al. 2022) and other grant‐funded research (e.g., Holtrop et al. 2018). Theater testing may also be used throughout the intervention adaptation process, depending on the proposed research questions. For example, Biello et al. (2021) used theater testing toward the end of app development to ensure usability, whereas we used theater testing early on to ensure LGBTQIA+ communities were a central part of the adaptation of EFCT.

Although we have applied theater testing to the cultural adaptation of an evidence‐based intervention, the flexible nature of theater testing may allow this method to be extended and generalized to other intervention research topics as well. For example, it may be possible to extend the use of theater testing to investigate engagement and retention. That is, participants could be exposed to intervention elements that are thought to influence engagement and retention outcomes, and then data could be gathered either through participant report (e.g., questions about factors that made them more or less likely to attend and/or come back to the theater testing session) and/or observation (e.g., percentage of participants who return to a follow‐up theater testing session) to inform subsequent adaptations to intervention engagement and retention strategies. In addition, although we have focused on demonstrating intervention‐specific elements in our theater testing examples (e.g., stages of EFCT), it is also conceivable that a theater test could feature nonspecific elements to obtain specific and informed participant feedback about other types of intervention or therapy domains, such as common factors. Broadly, the use of theater testing offers couple and family therapy researchers a useful method for engaging focal communities in the research process.
